# Thiamine deficiency activates hypoxia inducible factor-1α to facilitate pro-apoptotic responses in mouse primary astrocytes

**DOI:** 10.1371/journal.pone.0186707

**Published:** 2017-10-18

**Authors:** Kristy Zera, Jason Zastre

**Affiliations:** Department of Pharmaceutical and Biomedical Sciences, College of Pharmacy, University of Georgia, Athens, Georgia, United States of America; University of Kentucky, UNITED STATES

## Abstract

Thiamine is an essential enzyme cofactor required for proper metabolic function and maintenance of metabolism and energy production in the brain. In developed countries, thiamine deficiency (TD) is most often manifested following chronic alcohol consumption leading to impaired mitochondrial function, oxidative stress, inflammation and excitotoxicity. These biochemical lesions result in apoptotic cell death in both neurons and astrocytes. Comparable histological injuries in patients with hypoxia/ischemia and TD have been described in the thalamus and mammillary bodies, suggesting a congruency between the cellular responses to these stresses. Consistent with hypoxia/ischemia, TD stabilizes and activates Hypoxia Inducible Factor-1α (HIF-1α) under physiological oxygen levels. However, the role of TD-induced HIF-1α in neurological injury is currently unknown. Using Western blot analysis and RT-PCR, we have demonstrated that TD induces HIF-1α expression and activity in primary mouse astrocytes. We observed a time-dependent increase in mRNA and protein expression of the pro-apoptotic and pro-inflammatory HIF-1α target genes MCP1, BNIP3, Nix and Noxa during TD. We also observed apoptotic cell death in TD as demonstrated by PI/Annexin V staining, TUNEL assay, and Cell Death ELISA. Pharmacological inhibition of HIF-1α activity using YC1 and thiamine repletion both reduced expression of pro-apoptotic HIF-1α target genes and apoptotic cell death in TD. These results demonstrate that induction of HIF-1α mediated transcriptional up-regulation of pro-apoptotic/inflammatory signaling contributes to astrocyte cell death during thiamine deficiency.

## Introduction

Thiamine deficiency (TD) may occur in numerous conditions such as cancer and diabetes, or following bariatric surgery [1]. Additionally, it may occur in neurodegenerative disorders such as Alzheimer’s and Parkinson’s disease, although it is most prevalent in chronic alcoholics [2–5]. Wernicke-Korsakoff Syndrome (WKS) is a life-threatening consequence of TD occurring in up to 12.5% of chronic alcoholics [6]. It is characterized by memory loss, motor dysfunction and ocular disturbances resulting from focal damage in the thalamus and mammillary bodies [6]. Mechanistically, TD in alcoholism is multi-factorial involving poor nutrition, impaired coenzyme activation, and reduced intestinal absorption and renal transport [7–9]. It is estimated that ~30–80% of alcoholics have low circulating thiamine [10]. Although acute TD symptoms can be successfully treated by thiamine administration, untreated TD leads to irreversible lesions that are unresponsive to treatment [11].

A central feature in TD-induced neurological damage is severe deficits in cerebral metabolism through impairment of thiamine dependent enzymes [12, 13]. The resulting metabolic disruption leads to oxidative stress, inflammation, excitotoxicity, blood brain barrier dysfunction, cytotoxic edema, and apoptotic cell death [12, 14, 15]. Many effects of TD-induced metabolic disruption occur in astrocytes [16, 17]. TD disrupts neuron-astrocyte metabolic trafficking and induces focal lactic acidosis that further contributes to neurological damage [16]. Additionally, astrocyte-specific morphological changes are an early marker of TD-induced damage [18, 19]. Since astrocytes are essential to maintenance of neuronal energy and metabolism, astrocyte dysfunction may contribute to TD-induced neurological injury. However, the fundamental elements that initiate cell death as a consequence of TD are unresolved.

Recently, we have demonstrated that TD stabilizes Hypoxia Inducible Factor-1α (HIF-1α) in the absence of ischemic stress [20]. HIF-1α is a stress response transcription factor that regulates expression of genes with pro-inflammatory, pro-apoptotic and pro-survival functions [21]. The consequences of gene regulation by HIF-1α have been extensively investigated in ischemic stroke [22, 23]. Whether cells undergo apoptotic cell death or metabolic adaptation following ischemia is determined by both the severity of the insult and the cell type [23]. In neuron/astrocyte co-cultures exposed to hypoxia, HIF-1α expression mediated pro-survival responses in neurons, but pro-apoptotic responses in astrocytes [23]. Acute neuronal HIF-1α signaling promotes survival through metabolic reprogramming, erythropoiesis, and angiogenesis through expression of genes such as lactate dehydrogenase A (LDHA), glucose transporter-1 (GLUT1) and vascular endothelial growth factor (VEGF) [24–27]. Pro-apoptotic proteins such as BCL2/adenovirus E1B 19 kDa protein-interacting protein 3 (BNIP3) and BCL2/Adenovirus E1B 19kDa Interacting Protein 3 Like (Nix) are also established targets of HIF-1α and have been shown to induce apoptosis in an ischemic model [28, 29]. Other damaging responses such as inflammation have also been linked to chronic HIF-1α activation through direct up-regulation of Monocyte Chemoattractant Protein-1 (MCP1) [30–32]. Comparable histological lesions in patients with hypoxia/ischemia and TD have been described in the thalamus and mammillary bodies, suggesting a congruency between the cellular response to hypoxic and TD stress [33, 34]. Thus, stabilization and persistence of HIF-1α activity during TD may be a critical underlying initiator of apoptosis in astrocytes. It is unclear whether HIF-1α transcriptional activation during TD may contribute to neurological damage, as previously described in ischemia. Therefore, the purpose of this work was to determine whether HIF-1α activation during TD in mouse primary astrocytes may contribute to cell death through activation of pro-apoptotic proteins.

## Methods

### Isolation of primary mouse glial cultures

The animal protocol was approved by the University of Georgia Institutional Animal Care and Use Committee, and was compliant with Guidelines for the Use and Care of Laboratory Animals from the National Institutes of Health. Primary mouse astrocyte cultures were prepared from both male and female C57BL/6 mouse pups (Taconic Biosciences, Hudson, NY) as described by Schildge *et al*. with slight modifications [35]. Within 48h of birth, pups were euthanized and cortices were dissected, minced and digested with 0.25% trypsin and 2.21mM Ethylenediaminetetraacetic acid (EDTA) (Corning, Corning, NY) for 30min at 37°C. Trypsin was inactivated with 5mL RPMI 1640 media (Corning), and the cell suspension was centrifuged at 300xg for 5min at 4°C in an Allegra X-22R centrifuge (Beckman Coulter, Brea, CA). The supernatant was discarded, and pelleted cells were resuspended in RPMI 1640 media and subsequently filtered through a 100μm cell strainer (Greiner Bio-one, Monroe, NC). The filtered suspension was centrifuged at 300xg for 5min at 4°C in an Allegra X-22R centrifuge (Beckman Coulter). Supernatant was discarded and the pelleted cells were resuspended in 5mL RPMI 1640 media and subsequently filtered through a 40μm cell strainer (Greiner Bio-one). The filtered cell suspension was then seeded into RPMI 1640 media supplemented with 10% fetal bovine serum (FBS; Seradigm, Radnor, PA) and 0.1% gentamycin (Corning, Corning, NY) in an incubator at 37°C, 5% CO_2_, and 21% O_2_ at a density of ~3 brains in a T75 flask (Greiner Bio-one) with the media being refreshed after 24h. Mixed glia were maintained until they grew to full confluency, and then cultures were enriched for astrocytes by shaking at 180rpm in a Incubating Orbital Shaker (VWR, Radnor, PA) for 30min at 37°C followed by a media change and subsequent shaking for 6h at 260rpm [35]. Following enrichment, cells were passaged into a T175 flask until they reached confluency, and were subsequently passaged for treatments. Characterization of glial and astrocyte cultures was performed by both Western blot analysis and immunocytochemistry (S1 Fig).

### Cell culture treatments

Custom formulated thiamine deficient RPMI 1640 media supplemented with 10% fetal bovine serum (FBS), contains ~10nm thiamine (Data not shown). Therefore, this was not sufficient to achieve complete thiamine deficient conditions. While the use of dialyzed FBS may remove the majority of thiamine, other essential nutrients and growth factors are also lost. Therefore, thiamine deficiency was achieved using a thiamine pyrophosphate kinase-1 inhibitor, pyrithiamine hydrobromide (PT) (Sigma Aldrich, St. Louis, MO) which inhibits the conversion of thiamine to its active form, thiamine pyrophosphate [36]. While chemicals such as amprolium which inhibit thiamine transport may also induce thiamine deficiency, these have no effect on intracellular thiamine/thiamine pyrophosphate pools, which take long treatment periods to diminish [37]. Pyrithiamine treatment is commonly used to reproducibly induce a series of pathological changes both *in vivo* and *in vitro* [38]. Additionally, pathological changes associated with TD in primary astrocytes were observed following treatment with 10μM pyrithiamine, the concentration used in this study [39–41].

Confluent astrocyte-enriched cultures were seeded into custom formulated thiamine deficient RPMI 1640 media (United States Biological, Salem, MA) and refreshed with 10μM PT containing media every 48h during treatments up to 14 days. For thiamine repletion experiments, primary astrocytes were treated with 10μM pyrithiamine for 4d (PT). Subsequently, 3μM thiamine- containing RPMI 1640 was repleted into the culturing media for up to 5d (5R) without the presence of PT. To pharmacologically inhibit HIF-1α expression, 10μM YC1 was supplemented into PT containing media after a loading dose of 20μM for 24h. YC1 +/- pyrithiamine treatments lasted a total of 4d. A loading dose of YC1 was utilized prior to induction of thiamine deficiency to circumvent HIF-1α induction during simultaneous treatment with PT. Cisplatin treatment was used to demonstrate that the effects of YC1 treatment were specific to the inhibition of HIF-1α, without corresponding non-specific effects on apoptotic cell death. Primary astrocytes were treated with 30μM cisplatin +/- 10μM YC1 for 48h, following a loading dose of 20μM YC1 for 24h. In each treatment condition, control cells were maintained in RPMI 1640 media containing 3μM thiamine for the duration of treatment. Additionally, treatment start times were staggered such that each treatment period concluded at the same time. Therefore, all dishes were maintained equally in culture to control for changes in cell confluency and media conditions.

### Assessment of gene expression

Gene expression was assessed using quantitative real time PCR analysis. RNA was extracted using the E.Z.N.A. Total RNA Kit I (Omega Bio-Tek, Norcross, GA) following the manufacturer’s instructions. RNA was quantified using a Nanodrop 2000c Spectrophotometer (Thermo Scientific), and 1μg was reverse transcribed to cDNA with the qScript cDNA Synthesis Kit (Quanta BioSciences) following the manufacturer’s instructions. Changes in gene expression of the established HIF-1α target genes LDHA, GLUT1, and VEGF, as well as the pro-apoptotic genes BNIP3, Nix, Noxa and MCP1 were evaluated by qRT-PCR using a LightCycler 480 II (Roche Applied Science, Indianapolis, IN). Gene specific primers were designed using the Roche Universal Probe Library assay design center to correspond with a specific hydrolysis probe labeled at the 5’ end with fluorescein (FAM) listed in Table 1. Actin was used as a housekeeping gene, with probe and primer from the Roche Applied Science reference assay kit. Changes in gene expression were calculated using 2^-ΔΔCt^ method, with an assumed efficiency of 2 for relative quantification. Changes are expressed as the fold change relative to untreated samples.

**Table 1:** Primer sequences and probes from Roche Universal Probe Library used for RT-PCR analysis.

Gene	Primer Sequence	Probe
BNIP3	F: 5’-cctgtcgcagttgggttc-3’	#52
	R: 5’-gaagtgcagttctacccaggag-3’	
LDHA	F: 5’-ggcactgacgcagacaag-3’	#12
	R: 5’-tgatcacctcgtaggcactg-3’	
GLUT1	F: 5’-gaccctgcacctcattgg-3’	#99
	R: 5’-gatgctcagataggaatccaag-3’	
MCP1	F: 5’-catccacgtgttggctca-3’	#62
	R: 5’-gatcatcttgctggtgaatgagt-3’	
Nix	F: 5’-aacaacaactgcgaggaagg-3’	#70
	R: 5’-tagctccacccaggaactgt-3’	
Noxa	F: 5’-cattcctgatgaccacaacg-3’	#49
	R: 5’-tgctcaggaccctcttacaca-3’	
VEGF	F: 5’-aaaaacgaaagcgcaagaaa-3’	#1
	R: 5’-tttctccgctctgaacaagg-3’	

### Assessment of protein expression

To assess changes in protein expression and localization, primary astrocytes were harvested as whole cell lysates (WCL) or nuclear lysates for Western blot analysis as previously described [42]. WCL (50μg) and nuclear lysates (25μg) were resolved by electrophoresis on 12% SDS-PAGE gels and transferred to a polyvinylidene difluoride membrane. Membranes were blocked in 5% non-fat milk in tris buffered saline-tween 20 (TBS-T) for 3 h at 4°C. The membrane was immunoblotted for HIF-1α (GeneTex Cat# GTX127309 also ENCAB052WQI RRID:AB_2616089), HIF-2α (GeneTex Cat# GTX30123 RRID:AB_386038), LDHA (GeneTex Cat# GTX101416 RRID:AB_10726413), β-actin (Sigma-Aldrich Cat# A2228 RRID:AB_476697), p84 (GeneTex Cat# GTX70220 RRID:AB_372637), p53 (GeneTex Cat# GTX28590 RRID:AB_1241148), BNIP3L (Nix; GeneTex Cat# GTX111876 RRID:AB_2036357), Noxa (GeneTex Cat# GTX85521 RRID:AB_10725878), CCL2 (MCP1; GeneTex Cat# GTX48813 RRID:AB_11169950), Caspase-3 (GeneTex Cat# GTX110543 RRID:AB_10722709), Bax (GeneTex Cat# GTX61026 RRID:AB_10619997), Bid (GeneTex Cat# GTX22388 RRID:AB_368032), Poly ADP-ribose polymerase (Parp; GeneTex Cat# GTX26079 RRID:AB_367420), NeuN (GeneTex Cat# GTX30773 RRID:AB_1949456), Iba1 (GeneTex Cat# GTX100042 RRID:AB_1240434), GFAP (GeneTex Cat# GTX108711 RRID:AB_2037091), or BNIP3 (Abcam Cat# ab38621 RRID:AB_725737) for 24 h at 4°C (BNIP3, Nix, Noxa) or overnight at 4°C. Blots were washed 3 times each for 10 min in TBS-T, and then immunoblotted with 1:20,000 goat anti-mouse horseradish peroxidase (HRP)-conjugated secondary antibody (Millipore, Billerica, MA), 1:10,000 (BNIP3, Nix, Noxa) or 1:20,000 goat anti-rabbit HRP-conjugated secondary antibody (Bethyl Laboratories, Montgomery, TX) for 1 h at room temperature. Blots were visualized using Supersignal West Pico (Thermo Scientific, Rockford, IL) and captured with a Fluorchem HD2 digital imager (Alpha Innotech, San Leandro, CA). Densitometry was performed using Fluorchem SP software.

### APO-BrdU Tunel Assay

DNA fragmentation was assessed with the Apo-BrdU Tunel Assay Kit (Invitrogen, Carlsbad, CA) following the manufacturer’s instructions for both flow cytometry and microscopy applications. To analyze cells by flow cytometry, 1x10^6^ cells were fixed in 1% (w/v) formaldehyde in PBS and incubated on ice for 15 min. Cells were centrifuged at 300xg in an Allegra X-22R centrifuge (Beckman Coulter), washed with ice cold PBS and centrifuged again. Supernatant was discarded and cells were resuspended in ice-cold 70% (v/v) ethanol and incubated overnight at -20°C. Cells were centrifuged at 300xg in an Allegra X-22R centrifuge (Beckman Coulter) and washed with PBS to remove ethanol. Pelleted cells were resuspended in DNA-labeling solution supplied by the kit and incubated for 60min at 37°C with shaking every 15 min. Cells were rinsed in buffer supplied by the manufacturer and centrifuged at 300xg for 5 min in an Allegra X-22R centrifuge (Beckman Coulter). Cells were then incubated in antibody staining solution and incubated for 30 min at room temperature protected from light. Additionally, propidium iodide/RNase A staining buffer was added, cells were incubated for 30 min protected from light. Samples were analyzed by flow cytometry using a CyAn ADP analyzer (Beckman Coulter) with fluorescence emission at 530nm and 575nm. Data were analyzed using FlowJo v. 10 software (RRID:SCR_008520; FlowJo, LLC, Ashland, OR).

For the microscopy application, cells were grown on chamber slides (BD Falcon, Corning, NY) and fixed in 1% formaldehyde in PBS for 15 min at 4°C. Slides were washed twice with PBS and incubated in 70% ethanol at -20°C overnight. Fixed cells were stained with 17μM DAPI for 10 min protected from light, washed 3 times with PBS and incubated with DNA-labeling solution supplied with the kit for 60 min at 37°C. Cells were rinsed and stained with antibody staining solution supplied by the kit for 30 min at room temperature protected from light. Images were captured using an Axio Observer.A1 Inverted Microscope (Carl Zeiss Microscopy, Jena, Germany).

### Cell death detection ELISA

For quantitative determination of cytoplasmic histone-associated DNA fragments, the Cell Death Detection ELISA^PLUS^ (Roche LifeScience, Indianapolis, IN) was utilized following the manufacturer’s instructions. Briefly, cells were lysed in buffer supplied by the kit for 30 min at room temperature with shaking. Lysates were collected and centrifuged at 200xg for 10 min at 4°C in a Microfuge 22R Centrifuge (Beckman Coulter). Supernatant was added to the supplied microplate in duplicate along with Immunoreagent for 2h at room temperature with shaking at 300rpm. At the end of the incubation period, the solution was removed and plates were washed 3 times each with incubation buffer. Additionally, ABTS solution was added to each well and left for 10 min at room temperature with shaking at 250rpm. Finally, ABTS stop solution was added, and the absorbance at 405nm was measured using a SpectraMax M2 spectrophotometer (Molecular Devices; Sunnyvale, CA).

### Annexin V and propidium iodide (PI) staining

To assess phosphatidylserine translocation to the outer leaflet of the plasma membrane as a marker of apoptotic cell death, the FITC Annexin V/Dead Cell Apoptosis Kit (Invitrogen) was used following manufacturer’s instructions. Briefly, 1x10^6^ cells were harvested, washed in ice cold PBS and pelleted by centrifugation at 500xg in an Allegra X-22R centrifuge (Beckman Coulter). Cells were resuspended in 1X annexin-binding buffer supplied by the kit, and sample was stained for 15 min at room temperature. At the end of the incubation, cells were diluted in additional 1X annexin-binding buffer and kept on ice. Staining was analyzed by flow cytometry using a CyAn ADP analyzer (Beckman Coulter) with fluorescence excitation and emission at 530nm and 575nm, respectively. Data were analyzed using FlowJo v. 10 software (FlowJo LLC, Ashland, OR).

### Statistical analysis

All experiments were performed with three independent replicates unless otherwise stated. Statistical significance was evaluated among groups using a one-way analysis of variance with Tukey's post hoc test with a significance level of p<0.05 using GraphPad Prism 6^®^ (GraphPad Software; La Jolla, CA).

## Results

### Stabilization and activation of HIF-1α in thiamine deficiency

**Fig 1:**
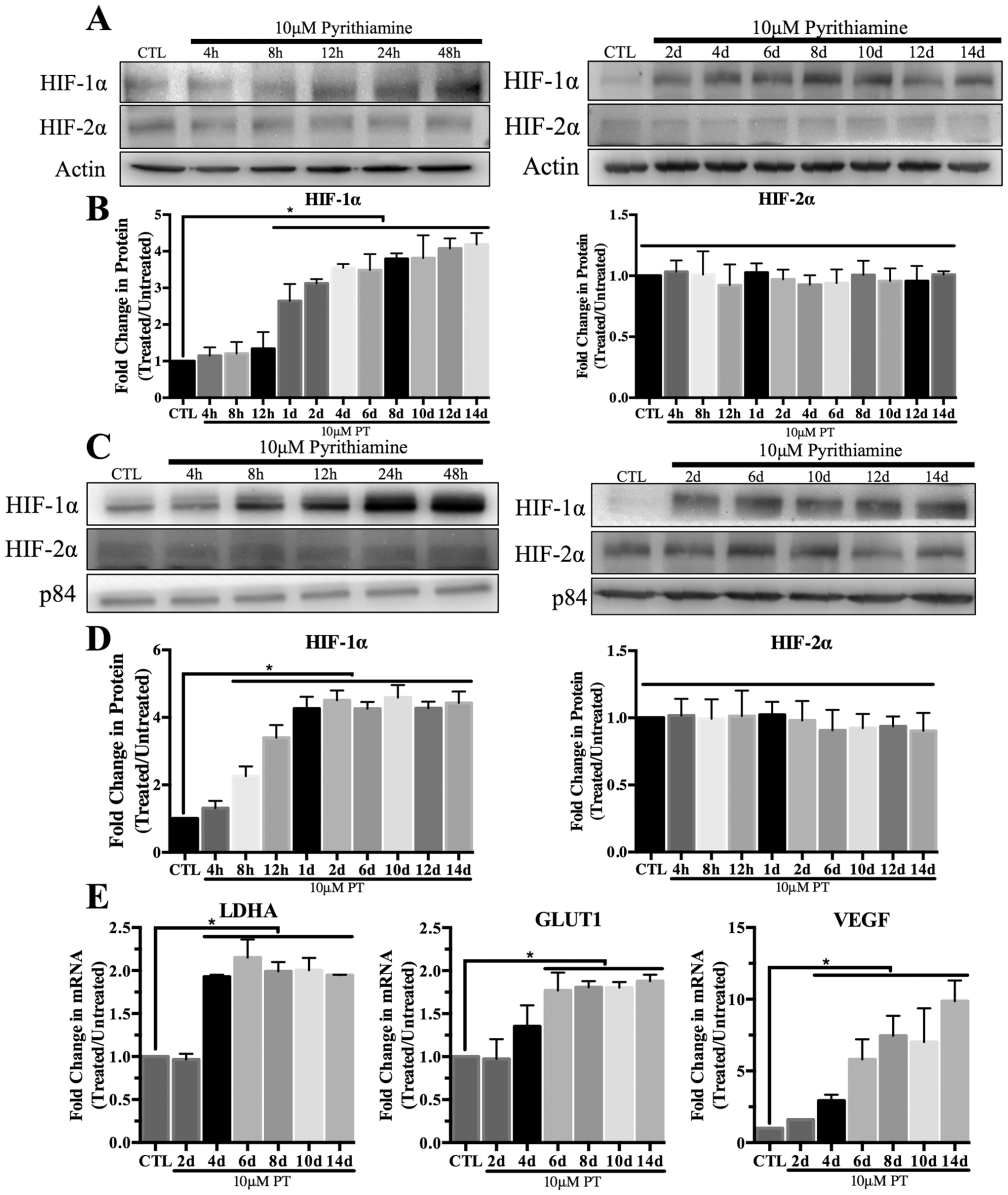
HIF-1α activation in mouse primary astrocytes. Cells were treated with 10μM pyrithiamine (PT) up to 14d to induce thiamine deficiency relative to 3μM thiamine control (CTL). Representative Western blots are shown for expression of HIF-1α in WCL (A) and nuclear lysates (C). Actin was used as a loading control for WCL while p84 was used for nuclear samples. Densitometry of mean protein expression +/- SD includes n = 3 independent experiments for (B) WCL and (D) nuclear lysates. E) Real time-PCR analysis of mRNA expression +/- SD of the established HIF-1α target genes LDHA, GLUT1 and VEGF. Data are normalized to Actin as a loading control and the control sample using the 2-^ΔΔCt^ method. (★) Represents a statistically significant difference of p<0.05 compared to CTL based on the results of a one-way ANOVA with Tukey’s post-hoc test.

Induction of thiamine deficiency in mouse primary astrocytes resulted in an ~3 fold increase in total HIF-1α expression as early as 24h of pyrithiamine (PT) treatment, which was maintained with minimal fluctuation in expression for up to 14 days (Fig 1A and 1B). Additionally, nuclear localization of HIF-1α increased as early as 8h after exposure to PT and was also maintained up to 14 days (Fig 1C and 1D). In contrast, no change in total protein or nuclear localization of HIF-2α was observed (Fig 1A–1D) In these same conditions, mRNA levels of the established HIF-1α target genes LDHA, GLUT1 was significantly increased ~2 fold (Fig 1E). Transcript levels of the target gene VEGF increased ~10 fold by day 14 (Fig 1E).

### Expression of pro-apoptotic proteins in thiamine deficiency

**Fig 2:**
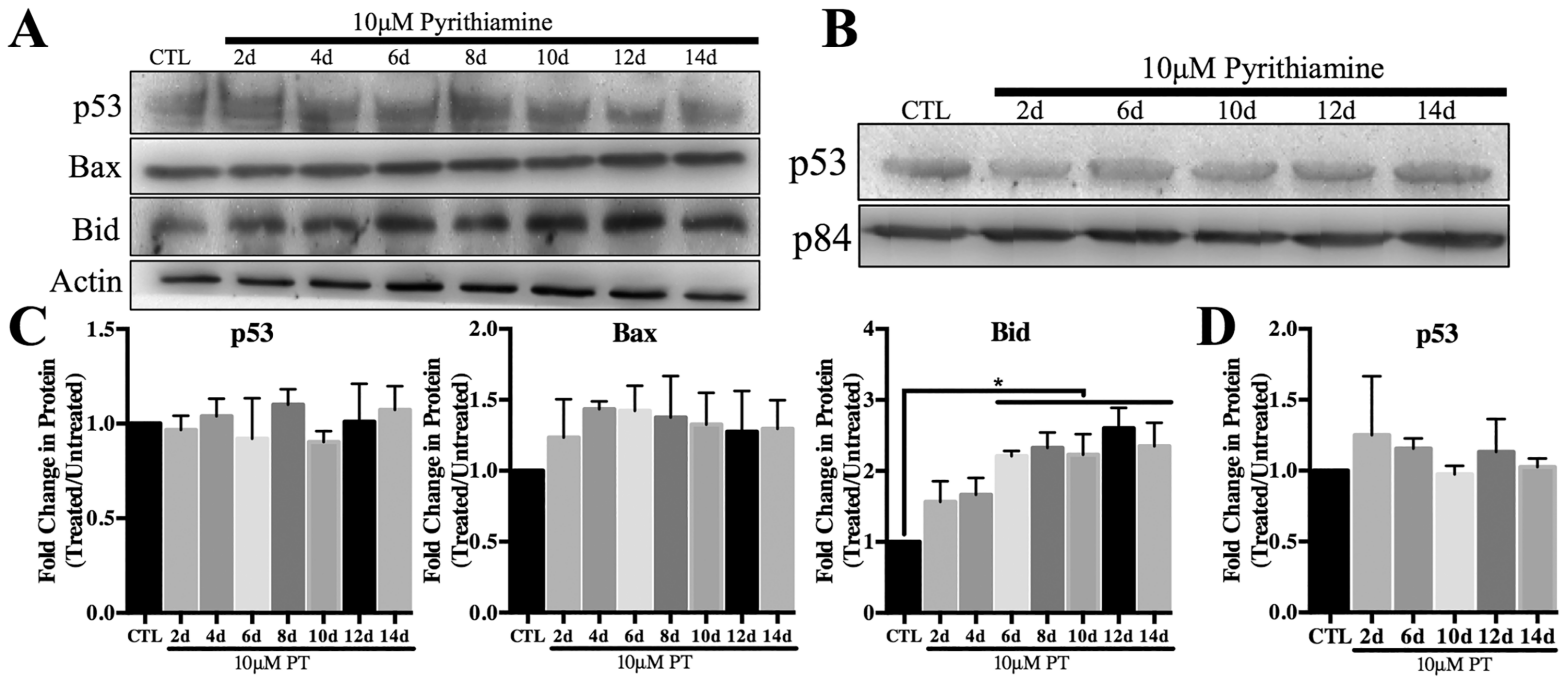
Effects of thiamine deficiency on the p53 pro-apoptotic pathway. Cells were treated with 10μM pyrithiamine up to 14d to induce thiamine deficiency compared to 3μM thiamine control (CTL). A) Representative Western blots are shown for expression of p53 and pro-apoptotic target genes Bax and Bid in WCL. Actin was used as a loading control. B) Representative Western blot of p53 nuclear localization in thiamine deficiency. P84 is used as a loading control. Densitometry of mean protein expression +/- SD includes n = 3 independent replicates for WCL (C) and nuclear lysates (D). (★) Represents a statistically significant difference of p<0.05 compared to CTL based on the results of a one-way ANOVA with Tukey’s post-hoc test.

**Fig 3:**
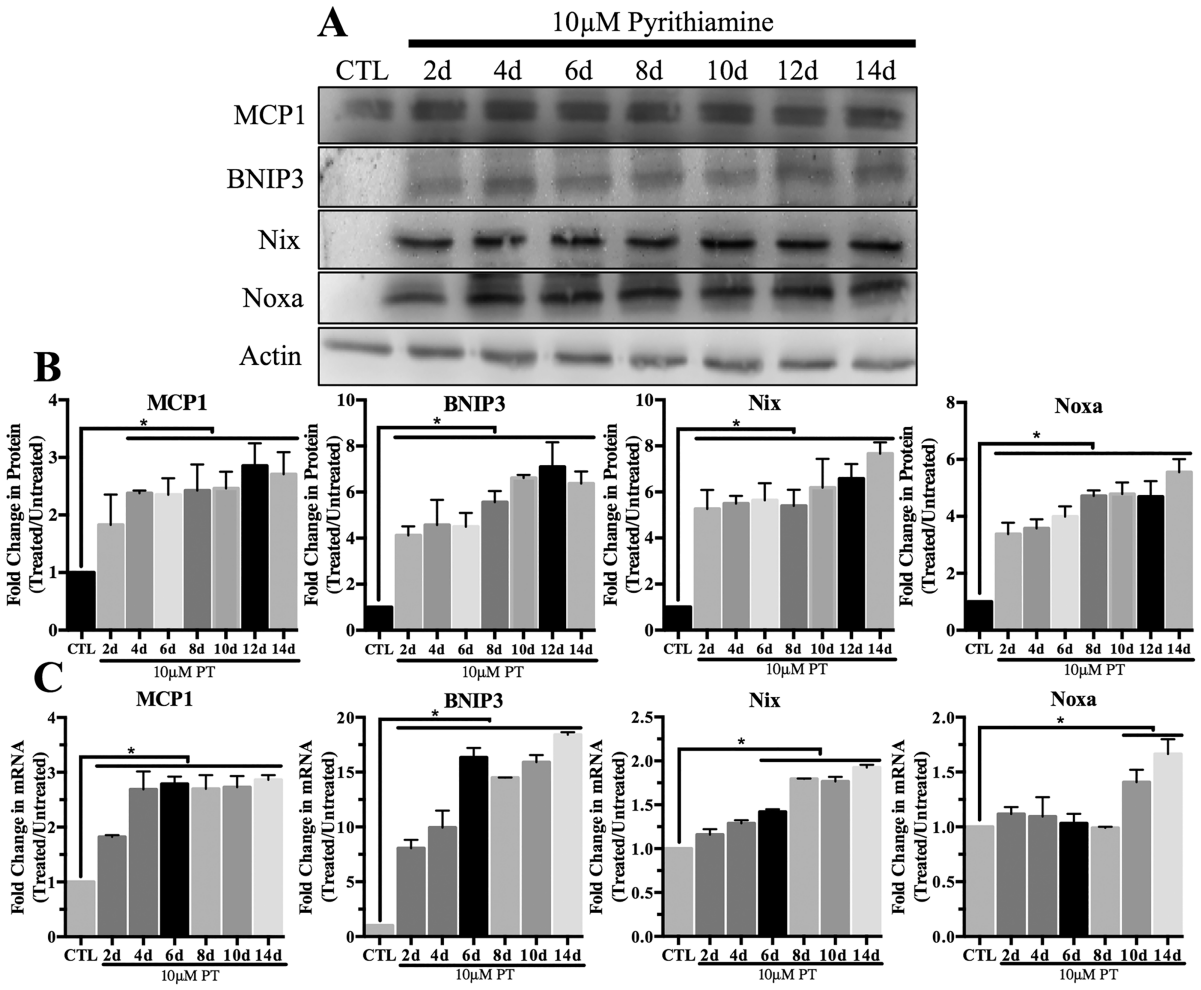
Effect of thiamine deficiency on expression of pro-apoptotic HIF-1α target genes. Primary astrocytes were treated with 10μM pyrithiamine up to 14d to induce thiamine deficiency compared to 3μM thiamine control (CTL). A) Representative Western blots are shown for expression of MCP1, BNIP3, Nix and Noxa in WCL. Actin was used as a loading control. B) Densitometry of mean protein expression +/- SD includes n = 3 independent replicates. C) Real time-PCR analysis of mRNA levels +/- SD of the HIF-1α target genes MCP1, BNIP3, Nix and Noxa. Data are normalized to Actin as a loading control and the control sample using the 2^-ΔΔCt^ method. (★) Represents a statistically significant difference of p<0.05 compared to CTL among n = 3 independent replicates based on the results of a one-way ANOVA with Tukey’s post-hoc test.

There was no observed change in total p53 expression (Fig 2A and 2C) or nuclear localization (Fig 2B and 2D) after 14 days of PT treatment. Protein expression of the pro-apoptotic p53 target gene Bax was not altered, while Bid expression significantly increased ~2.5 fold (Fig 2A and 2C). In contrast, Fig 3 demonstrates that both protein and mRNA expression of various pro-apoptotic HIF-1α target genes increased following PT treatment. Specifically, protein expression of the pro-inflammatory cytokine MCP1 increased ~3 fold, while pro-apoptotic BNIP3 protein levels increased up to 8 fold (Fig 3A and 3B). Similarly, protein levels of Nix increased up to 8 fold, while Noxa protein levels increased ~6 fold after 14 days of PT treatment (Fig 3A and 3B). Fig 3C demonstrates a significant increase in mRNA levels of MCP1, BNIP3, Nix and Noxa as compared to the untreated control.

### Thiamine repletion reduces HIF-1α mediated pro-apoptotic protein expression

**Fig 4:**
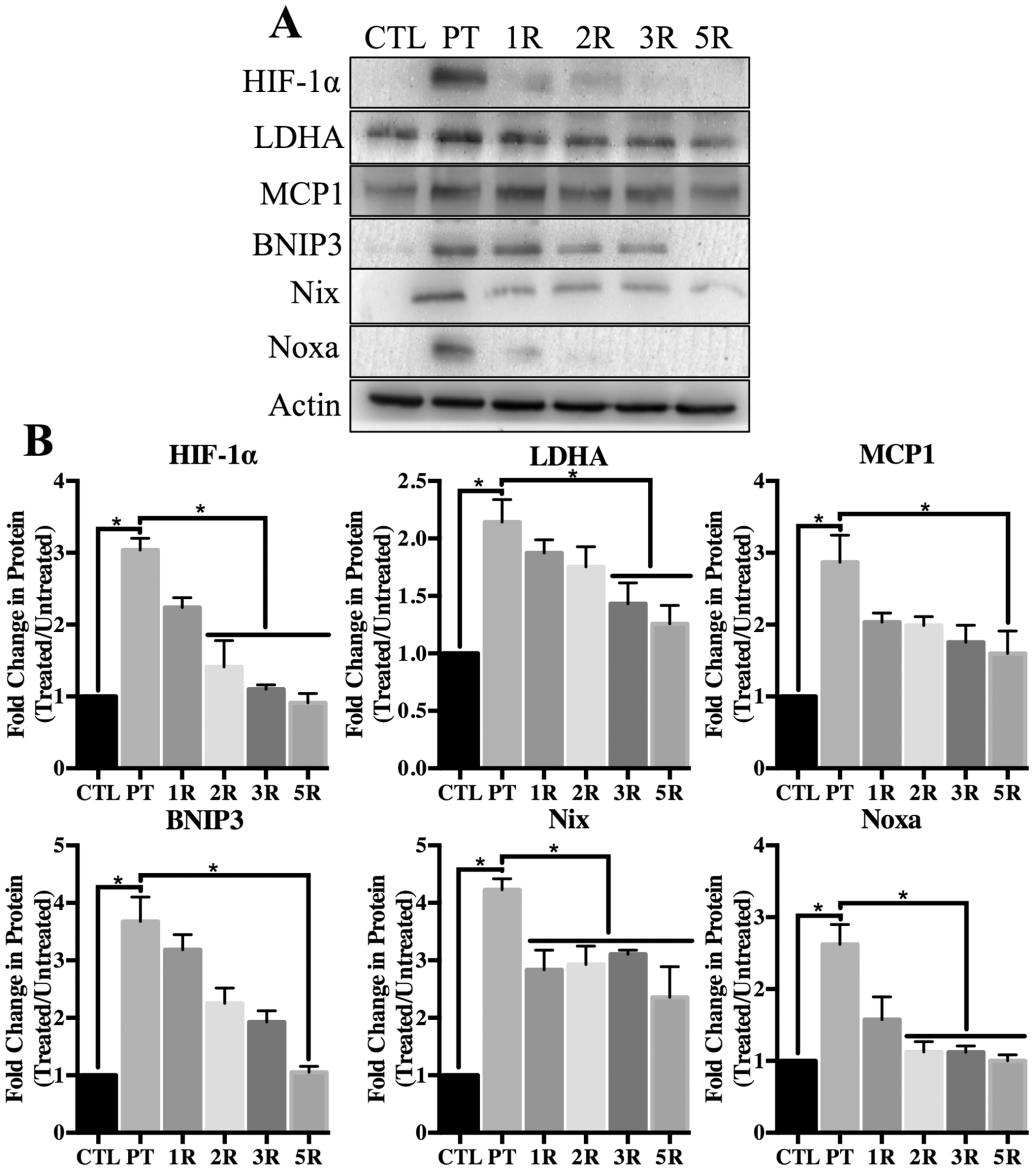
Effects of thiamine repletion on the expression of HIF-1α regulated pro-apoptotic proteins. Primary astrocytes were treated with 10μM pyrithiamine treatment for 4d (PT). Subsequently, 3μM thiamine was repleted into the culturing media for up to 5d (5R). A) Representative Western blot of HIF-1α and the established target gene LDHA in WCL. Expression of the pro-apoptotic target genes MCP1, BNIP3, Nix and Noxa in WCL are also shown. B) Densitometry of mean protein expression +/- SD is included with Actin as a loading control. (★) Represents a statistically significant difference of p<0.05 among n = 3 independent replicates based on the results of a one-way ANOVA with Tukey’s post-hoc test.

To achieve thiamine repletion following TD, astrocytes dosed with 10μM PT for 4 days were switched to normal (control) RPMI media containing 3μM thiamine (no PT) for up to an additional 5 days. Within 1 day of thiamine repletion following PT treatment a significant reduction in HIF-1α protein levels was observed that returned to control levels by day 5 of repletion (Fig 4A and 4B). The HIF-1α target gene LDHA was induced by thiamine deficiency, and subsequently reduced following thiamine repletion, although the protein did not return to control levels (Fig 4B). Furthermore, the expression of pro-apoptotic HIF-1α target genes was reduced following thiamine repletion. Noxa protein levels were significantly reduced following 24 h thiamine repletion, while BNIP3 protein was returned to control levels after 5 days repletion (Fig 4B). Although MCP1 and Nix protein levels were significantly reduced after thiamine repletion, they did not return completely to control levels after 5 days of repletion (Fig 4B).

### HIF-1α inhibition reduces pro-apoptotic protein expression

**Fig 5:**
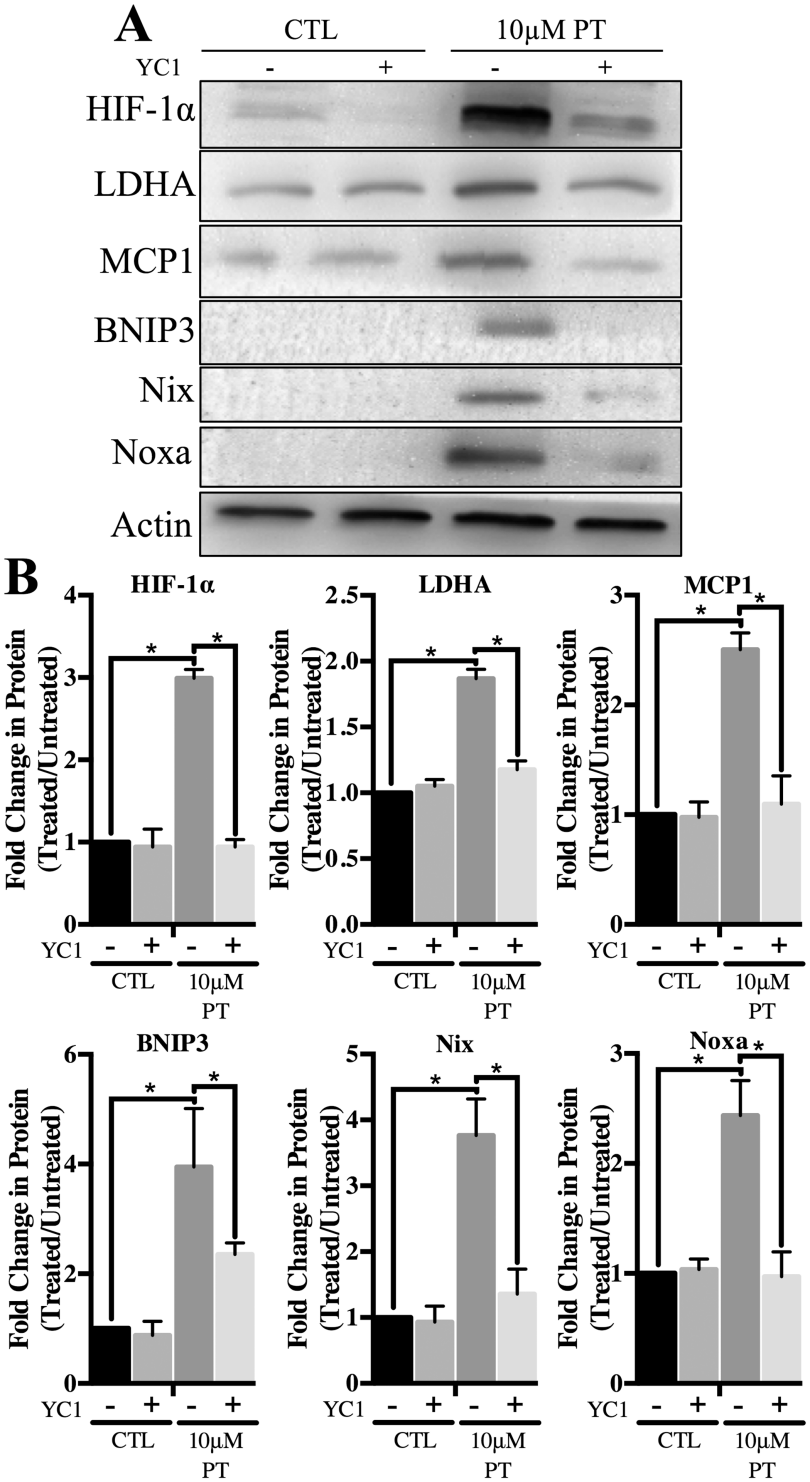
Effect of HIF-1α inhibition on expression of pro-apoptotic proteins. To achieve pharmacological inhibition of HIF-1α, 10μM YC1 was supplemented into PT containing media after a loading dose of 20μM for 24h. YC1 +/- pyrithiamine treatments lasted a total of 4d. A) WCL was assessed for expression of HIF-1α, LDHA, MCP1, BNIP3, Nix and Noxa. B) Densitometry of mean protein expression +/- SD is included with Actin as a loading control. (★) Represents a statistically significant difference of p<0.05 among n = 3 independent replicates based on the results of a one-way ANOVA with Tukey’s post-hoc test.

Pharmacological inhibition of HIF-1α was achieved using YC1, which reduces HIF-1α protein stabilization and transcriptional activity [43, 44]. After pre-treatment of astrocytes with a loading dose of 20μM YC1 for 24 h, 10μM YC1 was then supplemented into control (3μM thiamine) or PT containing media for a total of 4 days. In samples supplemented with 3μM thiamine and YC1, there was no significant change in expression of the proteins evaluated as compared to control samples (Fig 5A and 5B). In contrast, YC1 treatment significantly suppressed HIF-1α stabilization and attenuated protein expression of the HIF-1α target gene LDHA during PT treatment (Fig 5A and 5B). Additionally, YC1 was sufficient to minimize expression of pro-apoptotic HIF-1α target genes during PT treatment (Fig 5). A significant reduction in the protein expression of MCP1, BNIP3, Nix and Noxa was observed with PT and YC1 treatment compared to PT treatment alone (Fig 5A and 5B).

### Thiamine deficiency induced apoptosis is mediated through HIF-1α

**Fig 6:**
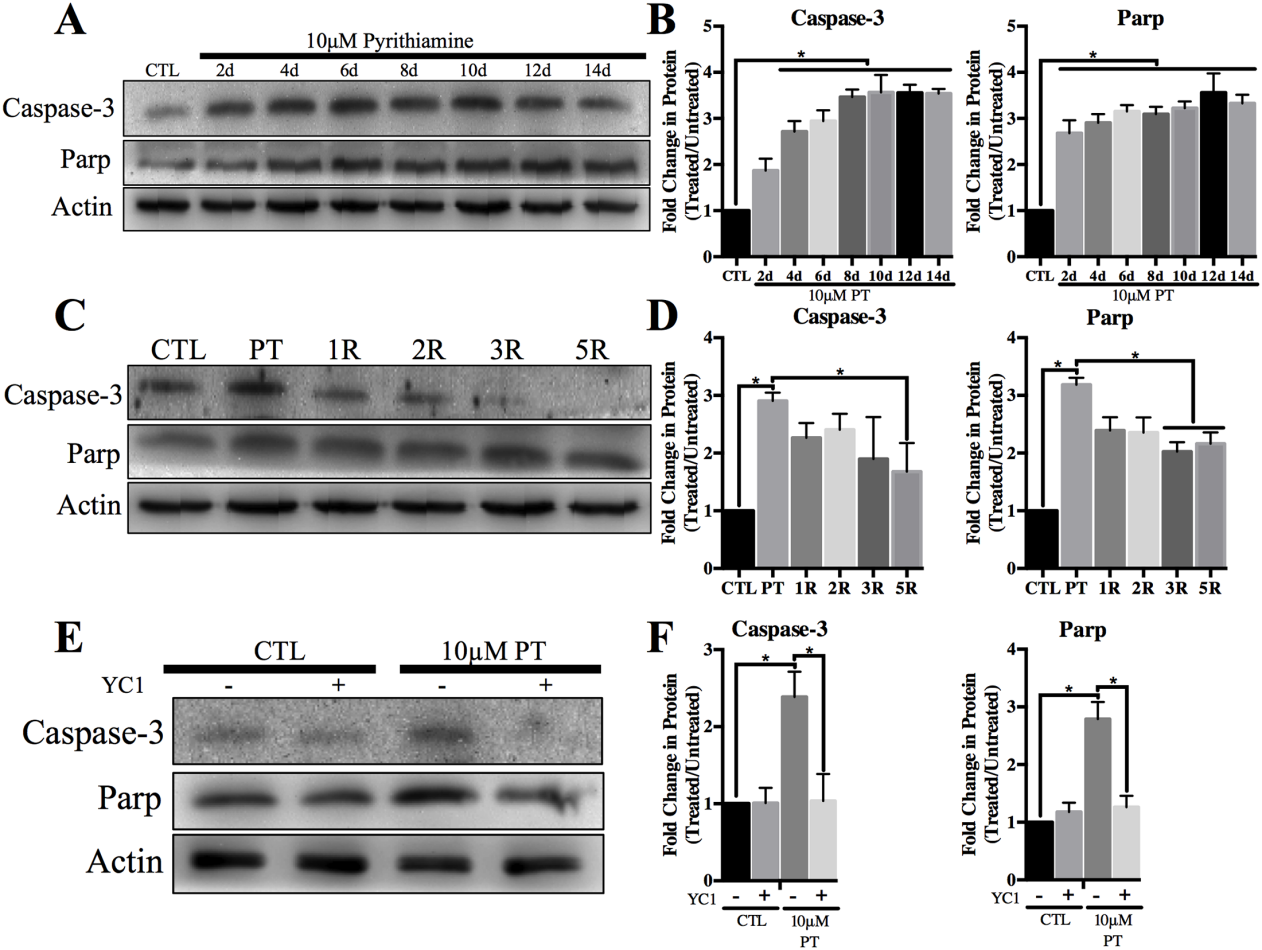
TD associated pro-apoptotic protein expression is reduced following inhibition of HIF-1α. Representative Western blots of cleaved Caspase-3 and cleaved Parp in WCL after A) treatment with 10μM pyrithiamine (PT) up to 14d, C) treatment with 10μM pyrithiamine for 4d with YC1 or E) treatment with 10μM pyrithiamine for 4d followed by 3μM thiamine repletion up to 5d (5R). Densitometry of mean protein expression +/- SD of each treatment set is shown with Actin as a loading control (B, D, F). (★) Represents a statistically significant difference of p<0.05 compared to CTL among n = 3 independent replicates based on the results of a one-way ANOVA with Tukey’s post-hoc test.

Thiamine deficiency induced expression of pro-apoptotic proteins that was inhibited by YC1 treatment and reduced following thiamine repletion (Fig 6). Cleaved Caspase-3 levels were significantly increased ~4 fold with PT treatment compared to the untreated control, while cleaved Parp expression was significantly increased ~3 fold (Fig 6A and 6B). Although Parp and Caspase-3 cleavage was significantly reduced, protein expression did not return to control levels following 5d of thiamine repletion (Fig 6C and 6D). Inhibition of HIF-1α activation through YC1 treatment was sufficient to block the cleavage of Caspase-3 and Parp (Fig 6E and 6F).

**Fig 7:**
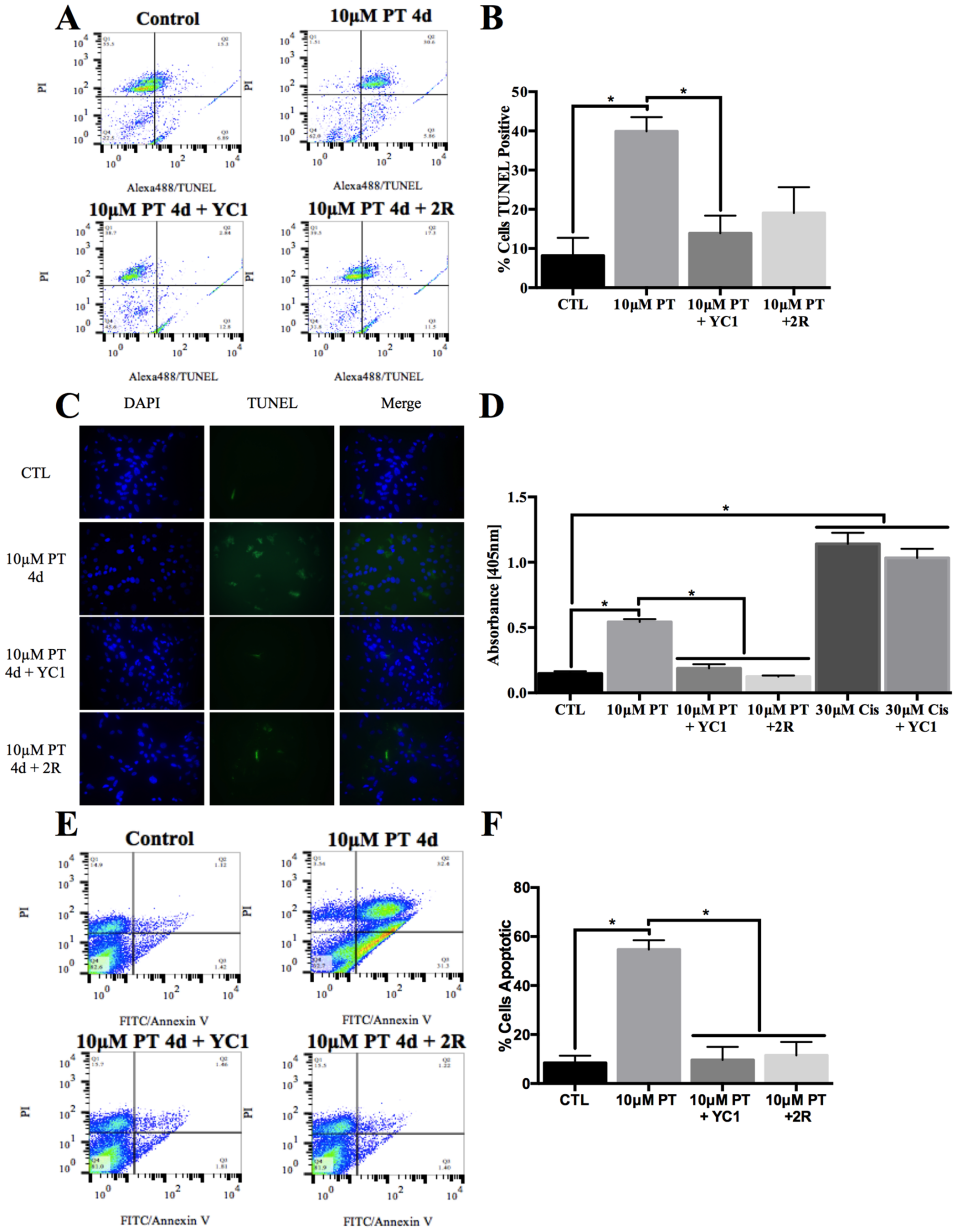
TD associated apoptosis is reduced following HIF-1α inhibition. Primary astrocytes were treated with 10μM pyrithiamine (10μM PT) for 4d, 10μM pyrithiamine for 4d with YC1 (10μM PT+YC1) or treatment with 10μM pyrithiamine for 4d followed by 3μM thiamine repletion for 2d (10μM PT+2R). Representative plots of TUNEL assay analyzed by flow cytometry (A) with a quantitative summary of n = 3 independent replicates +/- SD (B). C) Representative microscopy images of TUNEL assay performed on fixed cells are shown after treatment with PT for 4d, PT + YC1 for 4d or PT for 4d with 2d of repletion. D) N = 3 independent replicates of the Cell death ELISA +/- SD. E) Representative plots of PI/ Annexin V staining analyzed by flow cytometry with a summary of n = 3 independent replicates +/- SD (F). (★) Represents a statistically significant difference of p<0.05 compared to CTL among n = 3 independent replicates based on the results of a one-way ANOVA with Tukey’s post-hoc test.

DNA fragmentation was determined by a TUNEL assay where hydroxyl ends of damaged DNA were specifically labeled with dUTP. Fig 7A depicts representative plots generated by flow cytometry of all treatment groups subjected to the TUNEL assay. A shift of the cell population to the right side of the plot represents increased TUNEL staining, and therefore increased DNA fragmentation as seen in the PT treated group (Fig 7A). Quantification of TUNEL stained cells from each independent flow cytometry replicate reveals a statistically significant increase in DNA fragmentation in the PT treated group relative to control or YC1 treated cells (Fig 7B). Thiamine repletion for 2 days reduced DNA damage relative to PT treatment, although the change was not statistically significant (Fig 7B). Immunofluorescence images of the astrocytes subjected to the TUNEL assay visually confirm DNA fragmentation in PT treated cells, which was not observed following YC1 treatment or thiamine repletion (Fig 7C). Similarly, a significant increase in nuclear fragmentation into nucleosomal units was induced by PT treatment as demonstrated by the Cell Death ELISA^PLUS^ kit (Fig 7D). This effect was also significantly inhibited through YC1 treatment, and significantly reduced with thiamine repletion for 2 days (Fig 7D). Additionally, treatment with 30μM cisplatin for 48h was used to demonstrate that YC1 treatment did not reduce apoptosis without co-occurring inhibition of HIF-1α. Cisplatin treatment induced nucleosomal fragmentation, which was not significantly altered with simultaneous YC1 treatment (Fig 7E). Finally, Fig 7E demonstrates representative plots generated by flow cytometry of all treatment groups subjected to PI/Annexin V staining. A shift of the cell population to the right side of the plot represents increased Annexin V staining, and therefore increased phosphatidyl serine translocation to the outer leaflet of the plasma membrane as seen in the PT treated group (Fig 7E). Quantitation of flow cytometry replicates reveals that Annexin V staining was significantly increased following PT treatment, and significantly reduced following YC1 treatment and thiamine repletion (Fig 7F).

## Discussion

The consistency between TD and hypoxia/ischemia (H/I) is apparent with many cellular responses centralizing on HIF-1α activity. For instance, HIF-1α regulation of aquaporin-4 (AQP-4) and matrix metalloproteinase-9 (MMP-9) lead to edema and blood brain barrier (BBB) disruption during ischemia [45, 46]. Focal edema during TD also correlates with increased expression of AQP-4 and MMP9 [40, 47]. Neuronal expression of the HIF-1α regulated chemokine MCP1 is induced in both H/I and TD [32, 48, 49]. Our findings have for the first time directly correlated HIF-1α activation during TD to expression of pro-apoptotic proteins and subsequent apoptotic death in primary mouse astrocytes.

A related protein HIF-2α is also responsible for regulating the cellular hypoxic response. Although each protein recognizes the same consensus sequence, cell-type expression and activity of HIF-2α is significantly more restricted [50, 51]. We found that HIF-1α was stabilized in TD, however no induction of HIF-2α was observed. Both HIF-1α and HIF-2α are stabilized by the loss of prolyl hydroxylase (PHD) activity in H/I, although it appears in the context of TD that additional distinct regulatory mechanisms may be involved [22, 52]. Studies in renal carcinomas have demonstrated that BNIP3 expression correlates positively to HIF-1α activity, but negatively to HIF-2α suggesting distinct roles in apoptotic signaling as well [50]. Furthermore, a study in hepatocytes demonstrated a correlation between p53-mediated apoptotic death and HIF-2α expression, neither of which were observed in the primary mouse astrocytes during TD in this study [53].

H/I studies have demonstrated that acute HIF-1α activation is protective through increased oxygen availability due to the promotion of angiogenesis, and metabolic reprogramming to an aerobic shift [54, 55]. Direct HIF-1α trans-activation of VEGF mRNA results in increased angiogenesis and subsequent oxygen availability [56]. This is consistent with our work in mouse primary astrocytes, and previous studies in SK-N-BE cells which demonstrate an increase in VEGF mRNA during TD [20]. TD-mediated HIF-1α activation and up-regulation of VEGF may explain the clinical observation of high circulating VEGF and cerebral blood flow hyperperfusion observed in TD patients and rodent models [57–59]. HIF-1α signaling also attempts to preserve metabolism through regeneration of NAD+ via LDHA activity and maintenance of ATP production via rapid glycolysis [60]. HIF-1α mediated up-regulation of GLUT1 and LDHA promotes rapid glycolysis and reduces oxidative phosphorylation [25]. Likewise, up-regulation of GLUT1 and LDHA in mouse primary astrocytes as a direct consequence of HIF-1α activation is consistent with established metabolic sequalae during TD. The observed alterations to glucose metabolism result in impaired ATP production in astrocytes, and impact the metabolic coupling between astrocytes and neurons [61]. An increase in glucose utilization was found in TD animals that correlated with the onset of neuronal damage [62]. Additionally, lactate accumulation via LDHA activity is an established metabolic consequence of TD in astrocytes that also contributes to neuronal death and the formation of histological lesions [63]. Recent evidence suggests that lactic acidosis increases Aquaporin-4 protein expression and plasma membrane localization in astrocytes leading to cellular swelling, increased BBB permeability, and subsequent edema [64]. Additionally, astrocyte swelling is associated with increased glutamate release resulting in glutamate-mediated excitotoxicity which has been implicated in neuronal cell death in TD [65]. Glucose administration to TD patients further increases focal lactate production and LDHA expression in TD-vulnerable brain regions contributing to the precipitation and exacerbation of symptoms [66]. Overall this suggests that loss of astrocyte energetic integrity due to HIF-1α mediated metabolic reprogramming may contribute to neuronal injury and explain the clinical and biochemical manifestations of TD.

In contrast to its pro-survival function, HIF-1α mediated apoptosis is associated with chronic activation [25]. Apoptosis during H/I is related to HIF-1α trans-activation of the pro-apoptotic proteins BNIP3, Nix and Noxa [67, 68]. However, the increase in Noxa protein levels in astrocytes during TD did not appear to be linked to increased mRNA levels. Aside from transcriptional regulation, ubiquitination and proteasomal degradation of Noxa can be suppressed in response to cell stress [69]. Similar to our observations in TD, Noxa protein levels rapidly increased in response to ischemic stress, while the transcriptional response was delayed [67]. Further work is necessary to understand whether TD can suppress proteasome activity as a contributing factor to Noxa induction. Induction of BNIP3 and Nix expression induces plasma membrane permeability, mitochondrial membrane depolarization, pore opening, and increased ROS generation [68]. Consistent with H/I, cell death and expression of BNIP3, Nix and Noxa were attenuated by pharmacological inhibition of HIF-1α during TD in astrocytes. Noxa suppression was sufficient to rescue cells from H/I death, and decrease injury *in vivo* [67]. TD-induced apoptosis was suppressed by YC1 treatment, although the effects of thiamine repletion are less straightforward. Following thiamine repletion apoptotic cells are no longer detected, though it is unclear whether apoptosis is reversed to promote cell growth, or whether it merely halts the further progression of damage. YC1 is an established inhibitor of HIF-1α, although it was initially developed as a soluble guanyl cyclase (sGC) activator with effects on vasodilation. While studies have identified protective effects of YC1 through inhibition of glutamate-mediated toxicity, superoxide production, and pro-inflammatory activators, these are independent of sGC activation [70]. To date, limited studies have demonstrated anti-apoptotic effects of YC1 mediated through activation of the sGC pathway [71]. Our results demonstrate that YC1 had no impact on cisplatin induced apoptotic death, suggesting the reduction in apoptosis by YC1 during TD was mediated through HIF-1α inhibition. Therefore, pharmacological inhibition of HIF-1α may be a potential therapeutic target to limit TD-induced neurological damage. This strategy has already been investigated for treatment of H/I, and may therefore be adapted to TD related conditions [72].

Mechanistically, how TD induces HIF-1α may involve accumulation of pyruvate and lactate, ROS, or signaling through the PI3K/AKT pathway [73–75]. Lactate and pyruvate accumulation stabilizes HIF-1α in the absence of ischemia [74]. This is in contrast to canonical HIF-1α activation, which occurs in H/I due to PHD inactivation [76]. Hydroxylation of specific proline residues in the oxygen-dependent degradation domain of HIF-1α facilitates binding of the von Hippel Lindau protein which acts to ubiquitinate the protein for degradation [76, 77]. Due to structural similarities of pyruvate and lactate to α-ketoglutarate, a required cofactor for PHD activity, an abundance of these metabolites stabilizes HIF-1α [74]. While ischemic activation of HIF-1α occurs rapidly within 4h of treatment, our data demonstrates TD-induced HIF-1α stabilization within 8h [78]. While this is still a rapid response, the difference may reflect the time required to achieve loss of pyruvate dehydrogenase (PDH) activity and sufficient pyruvate accumulation. Reduced activity of the thiamine dependent enzyme PDH and up-regulation of LDHA during TD is consistent with increased pyruvate and lactate in patients [12, 79]. Alternatively, an increase in ROS generated by complex III of the electron transport chain (ETC) has been implicated in HIF-1α stabilization following H/I [75]. An abundance of ROS is a consequence of mitochondrial dysfunction in TD brains [11, 80]. A reduction in α-KGDH activity induces mitochondrial uncoupling and oxidative stress in astrocytes [38]. ROS-mediated HIF-1α activation limits flux through the ETC as a pro-survival response to combat oxidative stress [81]. Activation of the Akt/PI3K signaling pathway may also contribute to HIF-1α stabilization [73]. Akt/PI3K plays an important role in regulating nutrient homeostasis, and promoting cellular survival in nutrient deficient conditions [82]. Studies in breast cancer cells have observed no change in Akt total protein or phosphorylation following TD, suggesting this may not effect HIF-1α stabilization [83]. However, the role of Akt in TD still needs to be investigated in a primary cell model.

**Fig 8:**
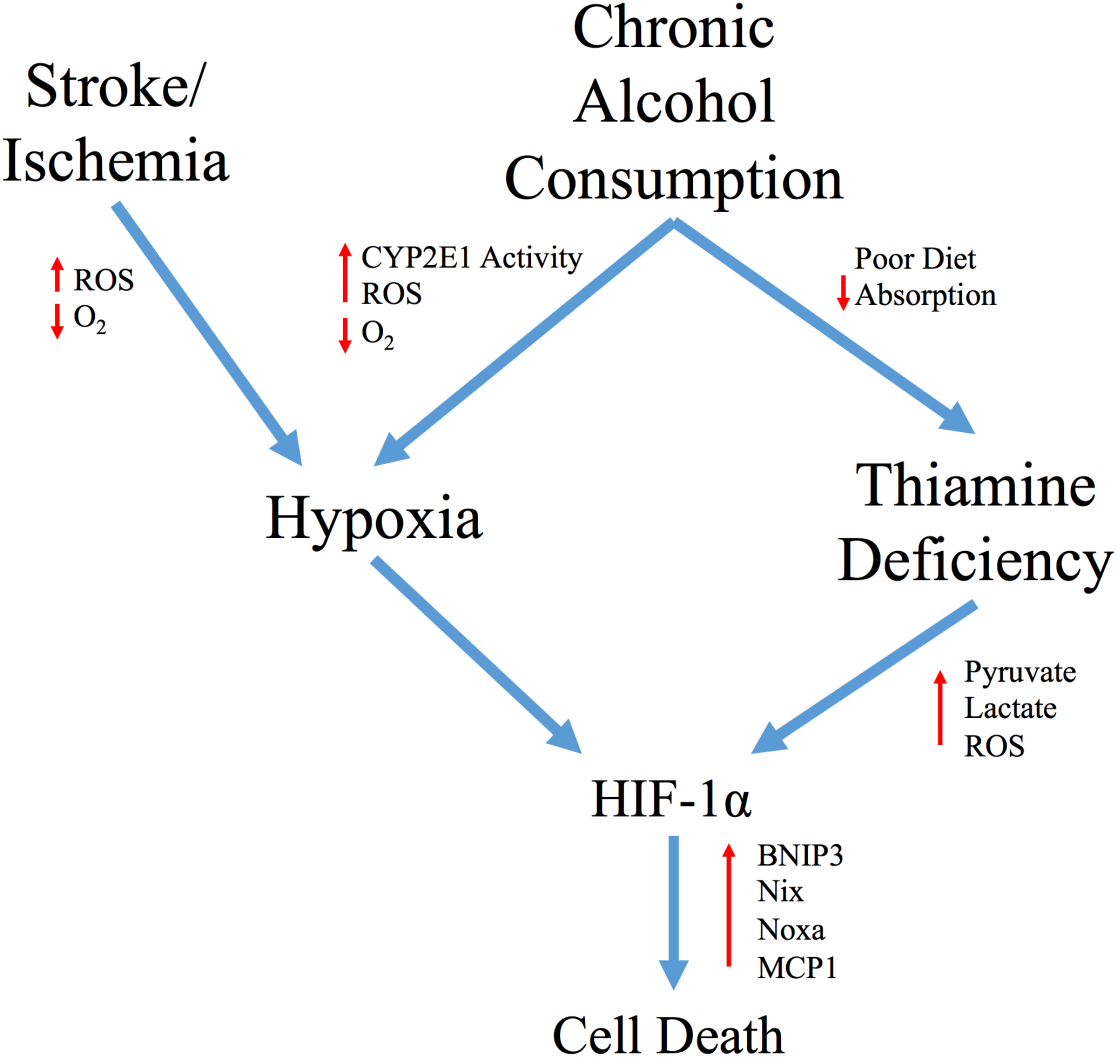
Schematic representation of the hypothesized role of HIF-1α in alcohol-induced neurological damage. The poor diet of chronic alcohol consumers and subsequent loss of intestinal thiamine transport contributes to TD in these patients. We have demonstrated that TD induces HIF-1α signaling and pro-apoptotic/inflammatory HIF-1α target genes such as MCP1, BNIP3, Nix and Noxa in astrocytes. Independent of TD, ethanol metabolism by CYP2E1 leads to an increase in oxygen consumption resulting in the development of a hypoxic microenvironment and an increase in ROS. In astrocytes, this may also lead to stabilization of HIF-1α and subsequent cellular death. Overall, this would suggest that apoptosis in either uncomplicated alcoholism or in conjunction with TD is mediated by a HIF-1α response to induce pro-apoptotic/inflammatory signaling, as observed in ischemic disease.

This work has identified a potential underlying transcriptional regulator that centralizes on many cellular responses occurring during alcohol and TD neurological injury (Fig 8). Interestingly, ethanol induced HIF-1α signaling is recognized as a mediator of Alcoholic Liver Disease [84–86]. In hepatocytes, CYP2E1 mediated ethanol metabolism increases oxygen consumption to induce a hypoxic microenvironment and produce ROS [87]. Pretreatment of mice with a HIF-1α inhibitor before ethanol ingestion correlated with a reduction in apoptosis, confirming an important contribution for HIF-1α mediated hepatotoxicity [85]. CYP2E1 has also been associated with ethanol mediated neurotoxicity [88]. High CYP2E1 expression in the hippocampus and cortex is observed in alcoholic patients and brain CYP2E1 was inducible after ethanol consumption in animal models [89, 90]. Increased HIF-1α mRNA and protein in the cortex have been observed in a rat model of chronic alcohol ingestion [91]. The concomitant induction of HIF-1α via ethanol metabolism and associated TD in multiple brain regions may provide insight into the increased progression of damage in rats fed both ethanol and TD diet compared to either alone [92]. Overall, this suggests that astrocyte cell death in either uncomplicated alcoholism or in conjunction with TD may centralize with a HIF-1α mediated transcriptional response and up-regulation of pro-apoptotic/inflammatory genes. Due to the important role for astrocyte integrity in neuronal survival, these findings suggest that TD-induced HIF-1α may contribute to neuronal cell death in TD. Moreover, along with thiamine administration, use of HIF-1α inhibitors such as YC1 may limit TD associated astrocyte death, similar to that following stroke.

## Supporting information

S1 Text Immunocytochemistry. (DOCX)

S1 Fig Characterization of cultures containing mouse primary astrocytes. A) Immunostaining of un-enriched glial cultures for GFAP, NeuN and Iba1. B) Immunostaining of GFAP, NeuN and Iba1 in primary astrocyte cultures. C) Western blot of whole brain tissue homogenates compared to cultures enriched for astrocytes. Expression of GFAP, NeuN and Iba1 was determined with Actin shown as a loading control.
(TIFF)
