# Supplementary material for: Thiamine deficiency activates hypoxia inducible factor-1α to facilitate pro-apoptotic responses in mouse primary astrocytes
Source: PLoS One. 2017 Oct 18;12(10):e0186707. doi: 10.1371/journal.pone.0186707 (PMC5646851; doi:10.1371/journal.pone.0186707)
Supplement: S1 Text — (DOCX) [file pone.0186707.s001.docx]

**Supporting Information:**

**Immunocytochemistry**

Primary astrocytes were grown to confluency in a 24 well plate (Greiner Bio-One) and fixed with 100% ice-cold methanol for 10 min at room temperature. Cells were blocked in 1mL of 1% bovine serum albumin (BSA) and 0.2% Triton X100 diluted in PBS for 1h at room temperature with light shaking. Cells were washed 3 times with ice cold PBS for 5 min each and incubated in the dark with 17μM DAPI for 10 min with shaking. Cells were washed 3 more times with PBS for 10 min each and incubated with primary antibody (1:500; NeuN, Iba1, GFAP) diluted in 1% BSA and 0.5% Tween 20 in PBS overnight at 4**°**C with shaking. Three more washes were performed in PBS for 10 min each and cells were incubated in secondary antibody (1:10,000) diluted in % BSA and 0.5% Tween 20 in PBS for 1h at room temperature with shaking. Images were captured using an Axio Observer.A1 Inverted Microscope (Carl Zeiss Microscopy, Jena, Germany).
