# Supplementary figures and images for: Thiamine deficiency activates hypoxia inducible factor-1α to facilitate pro-apoptotic responses in mouse primary astrocytes
Source: PLoS One. 2017 Oct 18;12(10):e0186707. doi: 10.1371/journal.pone.0186707 (PMC5646851; doi:10.1371/journal.pone.0186707)

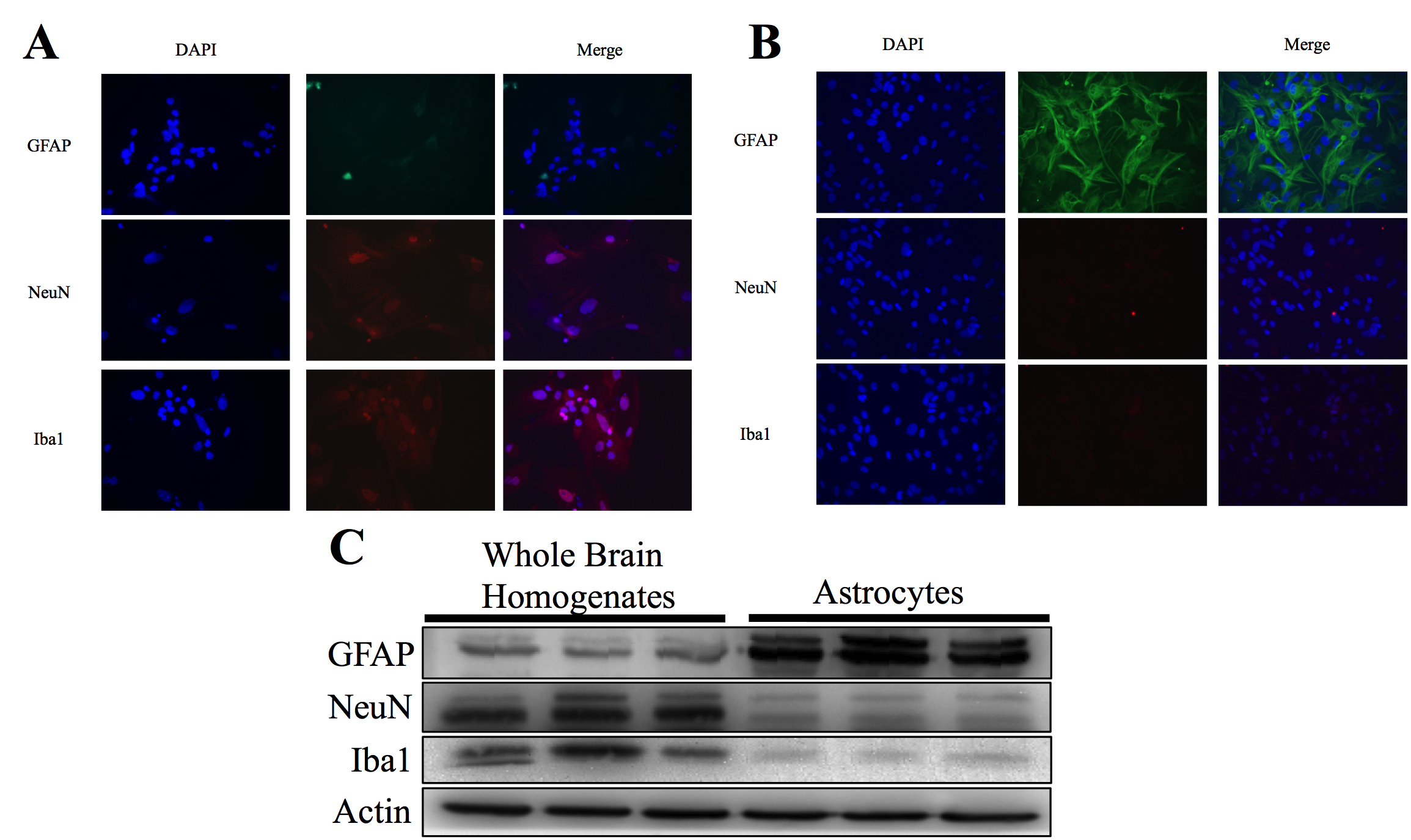

Supplement: S1 Fig — A) Immunostaining of un-enriched glial cultures for GFAP, NeuN and Iba1. B) Immunostaining of GFAP, NeuN and Iba1 in primary astrocyte cultures. C) Western blot of whole brain tissue homogenates compared to cultures enriched for astrocytes. Expression of GFAP, NeuN and Iba1 was determined with Actin shown as a loading control. (TIFF) [file pone.0186707.s002.tiff]
